# Endogenous retroviral solo-LTRs in human genome

**DOI:** 10.3389/fgene.2024.1358078

**Published:** 2024-03-28

**Authors:** Mingyue Chen, Xiaolong Huang, Chunlei Wang, Shibo Wang, Lei Jia, Lin Li

**Affiliations:** ^1^ National 111 Center for Cellular Regulation and Molecular Pharmaceutics, Key Laboratory of Fermentation Engineering, Hubei University of Technology, Wuhan, Hubei, China; ^2^ Department of Microbiology, School of Basic Medicine, Anhui Medical University, Hefei, Anhui, China; ^3^ Department of Virology, Beijing Institute of Microbiology and Epidemiology, Beijing, China; ^4^ State Key Laboratory of Pathogen and Biosecurity, Beijing, China

**Keywords:** human endogenous retrovirus, long terminal repeat, promoter, enhancer, long noncoding RNA, cancer

## Abstract

Human endogenous retroviruses (HERVs) are derived from the infection and integration of exogenetic retroviruses. HERVs account for 8% of human genome, and the majority of HERVs are solitary LTRs (solo-LTRs) due to homologous recombination. Multiple findings have showed that solo-LTRs could provide an enormous reservoir of transcriptional regulatory sequences involved in diverse biological processes, especially carcinogenesis and cancer development. The link between solo-LTRs and human diseases still remains poorly understood. This review focuses on the regulatory modules of solo-LTRs, which contribute greatly to the diversification and evolution of human genes. More importantly, although inactivating mutations, insertions and deletions have been identified in solo-LTRs, the inherited regulatory elements of solo-LTRs initiate the expression of chimeric lncRNA transcripts, which have been reported to play crucial roles in human health and disease. These findings provide valuable insights into the evolutionary and functional mechanisms underlying the presence of HERVs in human genome. Taken together, in this review, we will present evidences showing the regulatory and encoding capacity of solo-LTRs as well as the significant impact on various aspects of human biology.

## 1 Introduction

Human endogenous retroviruses (HERVs) are remnants of ancient retroviruses, which have infected and been integrated into the host genome (Weiss, 2006; Katzourakis et al., 2009). HERVs belong to transposable elements (TEs) based on the “copy and paste” life cycle of replicative transposition (Weiss, 2006; Katzourakis et al., 2009). A substantial number of HERVs have been retained in human genome during the long evolutionary history and constitute approximately 8% of human genome (Weiss, 2006). HERVs were previously considered “junk DNA” sequences (Weiss, 2006). Increasing findings suggest that HERVs play a crucial role in immune response, placental morphology, cancer development, and aging processes (Mi et al., 2000; Deniz et al., 2020; Wilson et al., 2020; Liu et al., 2023; Ng et al., 2023). For example, a recent study has demonstrated the presence of HERV-K (HML-2) envelope-reactive antibodies in a large proportion of patients with lung adenocarcinoma (LUAD) and the antibodies could promote lung cancer immunotherapy (Ng et al., 2023). It has been documented that syncytin is the envelope protein derived from HERVs and essential for placental morphogenesis (Mi et al., 2000). And emerging studies have further showed that HERVs participate in the expression regulation of placenta-associated genes (Frost et al., 2023). The effect of HERVs on cellular aging has also been discovered and the reactivation of the youthful HERV-K triggers cellular senescence and tissue aging (Liu et al., 2023). And HERVs have been proposed to serve as a potential target to alleviate aging (Liu et al., 2023). Apart from the function mentioned above, numbers of findings have suggested that HERVs are heavily implicated in occurrence and development of various cancers (Kitsou et al., 2023). One of classic examples is that the accessary protein Np9 is a critical molecular switch of multiple signaling pathways and could promote the growth of human leukemia stem/progenitor cells (Chen et al., 2013).

**FIGURE 1:**
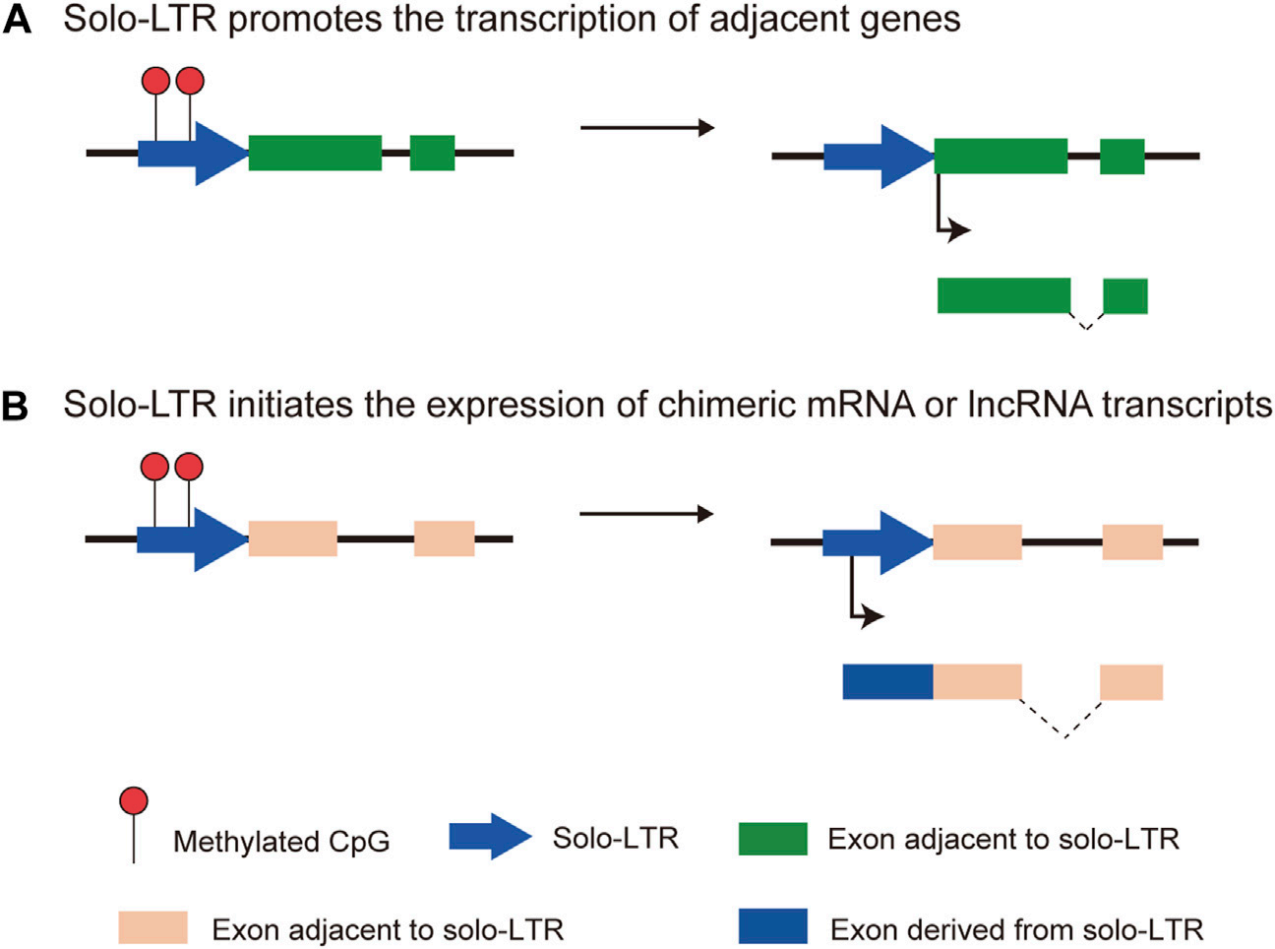
The two main functions of solo-LTRs. **(A)** Solo-LTR promotes the transcription of adjacent genes. **(B)** Solo-LTR initiates the expression of chimeric mRNA or lncRNA transcripts..

The complete proviral genome structure of HERVs consists of *gag*, *pro*, *pol*, and *env* genes flanked by long terminal repeats (LTRs), which usually serve as regulatory elements encompassing the TATA box and transcription factor binding sites (TFBSs) (Hurst and Magiorkinis, 2017). Due to the accumulation of numerous mutations, insertions and deletions (INDELs), most HERVs gradually lose the coding ability during evolution. Moreover, internal coding regions are deleted and the remaining LTR sequences eventually form solitary LTRs (solo-LTRs) through homologous recombination (Cohen et al., 2009). It has been reported that solo-LTRs are the most common HERV traces (Vargiu et al., 2016), accounting for about 90% of all HERV insertions (Subramanian et al., 2011; Thomas et al., 2018). The complete LTR elements comprise three conserved regions, namely U3, R and U5 (Vargiu et al., 2016). The U3 regions usually encompass promoter and enhancer elements that modulate the expression of internal genes (Vargiu et al., 2016). In most cases, the transcription initiation site (TSS) could be identified in the 5′ terminal R region while the 3′ terminal R region contains a polyadenylation (polyA) signaling site (Gifford and Tristem, 2003). Although solo-LTRs lose the internal genes, numerous studies have demonstrated the impact of solo-LTRs on the expression of adjacent genes (Ruda et al., 2004; Romanish et al., 2007; Conley et al., 2008; Cohen et al., 2009; Babaian and Mager, 2016). And solo-LTRs are also able to regulate the expression of distal genes as enhancers (Pi et al., 2004; Pi et al., 2010; Deniz et al., 2020). The exaptation of human endogenous retroviral promoters and enhancers has driven tissue-specific and lineage-specific patterns of gene expression and plays a substantial effect on diverse human biology processes, especially carcinogenesis and development (Conley et al., 2008; Cohen et al., 2009; Babaian and Mager, 2016; Hurst and Magiorkinis, 2017) (Figure 1A).

Various studies have documented the function of long non-coding RNAs (lncRNAs) in multiple biological processes including embryonic development, cell proliferation and immune response at the transcriptional and post-transcriptional levels (Statello et al., 2021). Emerging studies have revealed that active solo-LTRs could be transcribed and give rise to lncRNAs (Figure 1B), which are closely linked with multiple stages of tumorigenesis and metastasis (Prensner et al., 2013; Jin et al., 2019; Wu et al., 2020). These lncRNAs have been suggested as potential biomarkers for clinical cancer diagnosis and treatment (Prensner et al., 2013; Jin et al., 2019; Wu et al., 2020). For example, TROJAN, one solitary LTR70-derived lncRNA, promotes triple-negative breast cancer (TNBC) progression through ZMYND8 degradation (Jin et al., 2019).

In this review, we provide a window of the roles of solo-LTRs in human health and disease. Here we will illustrate the regulatory function of solo-LTRs demonstrated in a number of studies. Additionally, we will focus on the emerging discoveries of solo-LTRs-derived genes (mainly mRNAs and lncRNAs), exploring their expression patterns and regulatory mechanisms in diverse diseases. Importantly, these findings underscore the significance of the abundant reservation of solo-LTRs within human genome, particularly highlighting the potential involvement in cancer occurrence and progression.

## 2 Classification and nomenclature of HERVs

Based on phylogenetic evidence of *pol* gene, endogenous retroviruses (ERVs) have been classified into three main classes (Gifford and Tristem, 2003). Class I ERVs include gamma- and epsilonretroviruses, class II comprises alpha-, beta-, deltaretroviruses, and lentiviruses, and class III ERVs include spumaviruses (Gifford and Tristem, 2003). A specific tRNA hybridizes with the primer binding sites (PBS) of HERVs to initiate the reverse transcription reaction (Bannert and Kurth, 2006; Johnson, 2015). According to the identity of the 18 nucleotides in the PBS, HERVs are also classified into many families (Bannert and Kurth, 2006). For example, HERV-H family contains the PBS with a sequence similar to a histidine (H) tRNA, and those assumed to use tRNAHis would be also assigned to the HERV-H family (Bannert and Kurth, 2006). Thus, three ERV classes are grouped into diverse families (Bannert and Kurth, 2006; Johnson, 2015). Class I HERVs include HERV-H, HERV-W, HERV-E, HERV-F, HERV-I, HERV-P, HERV-T and so on. Class II HERVs have only one HERV-K family. And HERV-R and HERV-S are members of class III HERVs.

Due to the advent of next-generation sequencing technologies, several bioinformatics tools have been designed to detect polymorphic ERV insertions in human genome such as RetroTector (Sperber et al., 2007), RetroSeq (Keane et al., 2013) and ERVcaller (Chen and Li, 2019). Compared to existing tools such as RetroSeq, ERVcaller based on sequence alignment achieves both the higher sensitivity and precision for detecting simulated ERV and other TE insertions (Chen and Li, 2019). And ERVcaller can be applied broadly to other species other than human (Chen and Li, 2019). But the existing tools still have some limitations. For instance, ERVcaller requires ERV references (Chen and Li, 2019). Novel methods and softwares for identifying polymorphic ERVs are in need to be developed.

Databases describing the structure and distribution of HERVs have also been developed such as RepBase (Bao et al., 2015), Dfam (Storer et al., 2021) and HERVd (Paces et al., 2002). There are 770,551 HERV loci in human genome according to the annotation of RepeatMasker (http://repeatmasker.org) in UCSC table browser (https://genome.ucsc.edu/cgi-bin/hgTables) (Karolchik et al., 2004). The most common HERV traces are fragmented solo-LTRs while only 3,173 relatively intact HERV sequences were identified (Vargiu et al., 2016). It is an intriguing challenge to accurately classify the full spectrum of HERV insertions (particularly solo-LTRs) within related families.

As the high-throughput sequencing technology allows the obtaining of vertebrate genomes including a number of ERV sequences, a unified nomenclature system for ERV loci is urgently needed. A nomenclature system for retroviruses and ERVs proposed that the ERV loci should be composed of three elements, which include the lineage of retrovirus, a numeric ID and the host lineage (Gifford et al., 2018). For instance, the known full-length provirus HERV-K113, a member in HERV-K family (Jha et al., 2009), is designated as ERV-K(HML2).113-Hsa. This could contribute to the human genome annotation and the studies on pathophysiology function of HERVs.

## 3 Solo-LTRs acting as regulatory elements

**FIGURE 2:**
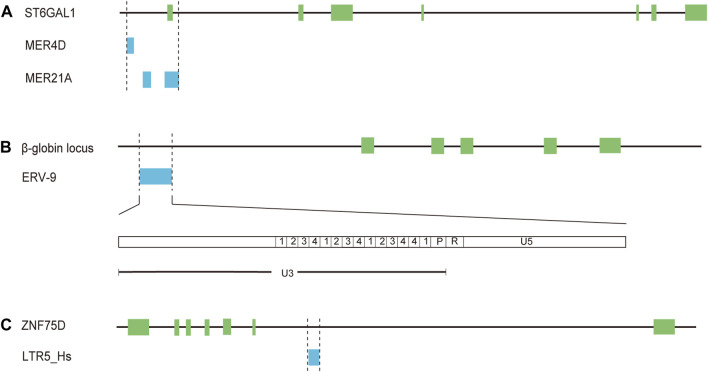
Solo-LTRs acting as regulatory elements for mRNAs. **(A)** MER4D and MER21A elements regulate the expression of ST6GAL1. **(B)** ERV-9 element regulates the expression of β-globin. **(C)** LTR5_Hs element regulates the expression of ZNF75D.

Numbers of studies have showed that HERVs are an abundant reservoir of promoters and enhancers that can initiate or regulate transcription of adjacent genes in human health and diseases especially various cancers and the majority of HERV regulatory sequences belong to LTR elements, many of which are solitary LTRs (van de Lagemaat et al., 2003; Conley et al., 2008; Cohen et al., 2009; Babaian and Mager, 2016). In addition to triggering the expression of novel genes, LTRs could function as alternative promoters different from the native promoters (van de Lagemaat et al., 2003; Conley et al., 2008; Cohen et al., 2009; Babaian and Mager, 2016). Several studies have also documented the cell-specific or tissue-specific transcriptional regulation of LTR elements (van de Lagemaat et al., 2003; Conley et al., 2008; Cohen et al., 2009). For example, expression of β-galactoside α-2,6-sialyltransferase I (ST6Gal1), a glycan-modifying enzyme mediating the attachment of α-2,6-sialic acids, is modulated by three different promoters, generating three mRNA isoforms, which have been identified in the liver, mature B-cells and a number of cell types, respectively (Aas-Eng et al., 1995; Milflores-Flores et al., 2012). Intriguingly, the expression of the transcript in mature B-cells is induced by a promoter, which includes several solo-LTRs (one MER4D element, two MER21A elements, and part of one MER21A element) (Lo and Lau, 1996) (Figure 2A, Supplementary Table S1). Further investigation showed that the solo-LTR sequences contain a number of known transcription factor binding sites (two AP2, two C/EBP and two NF-IL6) and the TATA box (Lo and Lau, 1996). It has been confirmed that the expression of ST6GAL1 in B cells is classically implicated in the dysregulated B cell development and immunoglobulin levels of ST6GAL1-deficient mice (Irons et al., 2020). So these solo-LTRs may play an important role in immune regulation through modulating the transcription of ST6GAL1. Another known example of the LTR with tissue-specific promoter and enhancer activities is LTR L47334, a solitary HERV-K LTR (Ruda et al., 2004). The promoter activity of the LTR was relatively weak in renal cell carcinoma GS and acute T cell leukemia Jurkat cells while a high promoter activity was detected in Tera-1 cells (Ruda et al., 2004). The enhancer activity of the LTR was only detected in Tera-1 cells and not in closely related NT2/D1 cells (Ruda et al., 2004).

The enrichment of multiple TFBSs in LTR elements is critical for transcriptional regulatory networks and phenotypic heterogeneity. A previous study has revealed that a significant proportion (32.1%) of the tumor suppressor protein p53 binding sites are embedded in LTR elements of ERV1 superfamily (LTR10B1, LTR10E, MER61E, LTR10D, and MER61C) (Bourque et al., 2008). For LTR9B elements from ERV1 superfamily, 33.2% of these repeats are bound by the key regulatory protein OCT4 in human embryonic stem cells (Kunarso et al., 2010). One of the notable examples is the solitary LTR of ERV-9 family. The LTR is located upstream of the human β-globin locus (Yu et al., 2005) (Figure 2B, Supplementary Table S1). The U3 region of the LTR element contains 14 tandemly repeated subunits with recurrent CCAAT and GATA motifs (Yu et al., 2005) (Figure 2B). The CCAAT motif could bind the ubiquitous transcription factor NF-Y while the GATA motif binds the GATA family of transcription factors, including GATA-1 and -2 (Yu et al., 2005). NF-Y bound at the CCAAT motif recruits GATA-1 and -2, thus generating a novel LTR enhancer-pol II complex (Yu et al., 2005; Hu et al., 2017). Furthermore, synthesis of lncRNA from the 5′ end of the R region through the U5 region into the downstream genomic DNA is initiated by the LTR enhancer complex (Hu et al., 2017). The lncRNA could act in cis to modulate long-range LTR enhancer function to activate transcription of downstream genes critical to erythropoiesis (Hu et al., 2017). In addition, about 80% of the LTRs among the 50 most highly expressed HERV-H proviruses are bound by core transcription factors associated with pluripotency, including POU class 5 homeobox 1 (OCT3/4), sex determining region Y-box 2box 2 (SOX2), and NANOG homeobox (NANOG) (Ohnuki et al., 2014). It has been demonstrated that LTR7, the LTR element of HERV-H, could promote cell reprogramming toward induced pluripotent stem cells (iPSCs) through being bound and activated by OCT3/4, SOX2, and KLF4 (Ohnuki et al., 2014). Therefore, LTR elements play a key role in expanding the repertoire of TFBSs and allowing a fine regulation of cellular biological processes.

It has been inferred that HERVs likely exert a slight effect on gene expression (Cohen et al., 2009). But to our knowledge, HERVs, notably LTR elements, contribute greatly to the regulatory networks of human genes. Much research efforts are dedicated to describe the effect of LTR-derived regulatory elements on human diseases especially cancers, and multiple findings have been reported including inducing the expression of oncogenes (Lamprecht et al., 2010; Nguyen et al., 2019), triggering the transcription of HERVs-derived double-stranded RNAs (dsRNA) associated with immune response (Zhou et al., 2021), and modulating the expression level of adjacent transcription factors (Ito et al., 2020; Zhou et al., 2021). In addition, there are increasing evidences that LTRs could be derepressed and play a role in shaping placental development (Yu et al., 2023), favoring immune response and driving human evolution (Jung et al., 2017; Srinivasachar Badarinarayan et al., 2020).

### 3.1 Breast cancer

Breast cancer is the most prevalent malignancy among women with its mortality rate ranking second only to that of lung cancer, as indicated by a recent study (Siegel et al., 2022). Numerous studies have demonstrated that HERVs, particularly HERV-K, exhibit upregulation in breast cancer, producing the relative mRNAs and proteins (Seifarth et al., 1998; Wang-Johanning et al., 2001; Wang-Johanning et al., 2008; Rhyu et al., 2014). For instance, Env protein can serve as the tumor-associated antigen to elicit immune responses from T cells and B cells (Wang-Johanning et al., 2008). Further investigations have revealed that solo-LTRs could be also implicated in occurrence and development of breast cancer through acting as promoters or enhancers (Nguyen et al., 2019). LTR5HS is an ape-specific LTR class of HML-2 (Fuentes et al., 2018). Two LTR5HS elements with 99% homology have identical progesterone response elements (PREs) and the octamer-binding transcription factor 4 (OCT4) binding motifs, and are located in the intron regions of SLC4A8 and IFT172 respectively (Nguyen et al., 2019) (Supplementary Table S1). Both of the two LTR5HS elements could activate the transcription of downstream sequences, producing antisense RNAs against SLC4A8 and IFT172 respectively (Nguyen et al., 2019). And the responsiveness to sex hormones could be completely abolished if the OCT4 motif of LTR5HS is deleted in breast cancer T47D cells (Nguyen et al., 2019). Methylated PRE of LTR5HS binds progesterone receptors (PRs) with higher affinity in T47D cells, and PRs would recruit OCT4 to the LTR5HS element, thus activating the expression of HERV-K, the Env protein of which promotes cancer development through the Ras-Raf-MEK-ERK pathway (Nguyen et al., 2019). These results have demonstrated the crucial role of LTR5HS elements in regulating host gene expression and enhanced our understanding of the crosstalk between the sex hormones and HERVs in breast cancer.

A recent study has showed that activation of p53 with the MDM2 inhibitor (a key negative regulator of p53) promotes the expression of HERVs-derived dsRNAs in breast cancer cells, thereby inducing the interferon pathway (Zhou et al., 2021). Further investigation has suggested that the expression of HERVs-derived dsRNAs is resulted from increased occupancy of p53 on HERV promoters (such as LTR26E) and inhibition of two major HERV repressors, histone demethylase LSD1 and DNA methyltransferase DNMT1 (Zhou et al., 2021). In contrast to the prior studies, the findings offer novel mechanistic insight into HERVs-mediated enhancement of anti-tumor immunity (Zhou et al., 2021).

### 3.2 Lung cancer

Lung cancer, the second most prevalent cancer, has the highest death rate among all malignant tumors in the United States (Siegel et al., 2022). The abnormal expression of RNA and protein products of HERV-K, HERV-H and other HERV families has been previously documented, and HERVs are suggested to be potential biomarkers for the diagnosis of lung cancer (Zare et al., 2018; Yang et al., 2022). Two insertionally polymorphic solo-LTRs termed HML-2_sLTR (1p13.2) and HML-2_sLTR (19q12) were identified in tissues of lung cancer, and the two LTRs include transcriptional regulatory regions such as a putative TATA box, polyadenylation signal, initiator sequence and enhancer core (Kahyo et al., 2013). A prevalence of HML-2_sLTR (1p13.2) homozygosity was detected in female never-smoking lung adenocarcinoma patients aged 60 years and over, suggesting that HML-2_sLTR (1p13.2) is related to the susceptibility to LUAD in female never-smokers in an age-dependent manner (Kahyo et al., 2013). Acidic mammalian chitinase (CHIA) is located in the vicinity of HML-2_sLTR (1p13.2) and could inhibit lung epithelial cell apoptosis (Kahyo et al., 2013). It has been speculated that HML-2_sLTR (1q13.2) with age-dependent changes of CpG methylation level may modulate the expression of CHIA as an enhancer, and thus excessive CHIA levels could facilitate the carcinogenesis of LUAD in patients aged over 60 years through anti-apoptotic mechanism (Kahyo et al., 2013).

Krüppel-associated box (KRAB) domain-containing zinc-finger family proteins (KZFP) are one of the largest groups of transcription factors in tetrapods (de Tribolet-Hardy et al., 2023). It has been reported that the expressed HERVs in tumors (such as LTR70, LTR25, LTR5B, and LTR5_Hs) are predominantly in the vicinity of TSSs of KZFP genes and some of these HERVs could act as enhancers modulating the expression of KZFP genes in LUAD cells (Ito et al., 2020). For instance, CRISPR-Cas9 excision of a solitary LTR MER21B resulted in a decreased expression of adjacent genes including KZFP genes (Ito et al., 2020). The expression of zinc finger protein ZNF75D was diminished upon CRISPR-Cas9-mediated knockout of LTR5_Hs in LUAD cells (Ito et al., 2020) (Figure 2C, Supplementary Table S1). Moreover, the expression status of KZFPs and HERVs has been shown to be associated with survival status in LUAD, urothelial bladder carcinoma (BLCA), head-neck squamous cell carcinoma (HNSC), and kidney renal papillary cell carcinoma (KIRP) (Ito et al., 2020). These results raise a possibility that LTRs may be released from the normal repressive mechanisms and then promote the occurrence or development of lung cancer as regulatory elements.

### 3.3 The impact of solo-LTRs on placental development

It is well known that syncytin, the envelope of an endogenous defective retrovirus HERV-W, plays vital role in human placental morphogenesis (Mi et al., 2000). And increasing studies have indicated that HERVs especially LTRs also participate in placental development in other ways such as acting as regulatory elements (Cohen et al., 2009). A recent study has further revealed that numbers of HERVs (nearly all of which are primate-specific) could function as promoter or enhancer with dynamic changes during differentiation of human trophoblast (Frost et al., 2023). And integration of the HERVs might be associated with placental evolution across primates (Frost et al., 2023). LTR8B and LTR10A, belonging to ERV1 superfamily, exhibit enhancer activity in differentiated placental trophoblasts (Frost et al., 2023). Notably, LTR8B demonstrates a remarkable ability to enhance the expression of PSG5 (Supplementary Table S1), one of the most highly expressed human pregnancy-specific glycoproteins (PSGs), following differentiation into extravillous trophoblasts (EVT) and syncytiotrophoblasts (SynT) (Frost et al., 2023). Two solitary LTR10A elements located in introns of endoglin (ENG) and placenta-specific CSF1R transcripts respectively could promote the expression of the two genes as enhancers in both EVT and SynT cell pools (Frost et al., 2023) (Supplementary Table S1). CSF1/CSF1R signaling could promote the growth, proliferation and migration of human trophoblast (Frost et al., 2023), and soluble ENG has been reported to be involved in the pathogenesis of preeclampsia by inducing endothelial cell dysfunction (ten Dijke et al., 2008). Another recent study has demonstrated that several LTR families (such as MER41A, MER50 and LTR8B) adjacent to genes upregulated specially in SynT tend to have increased H3K27ac and decreased H3K9me3 occupancy in SynT relative to human trophoblast stem cells (hTSCs) (Yu et al., 2023). MER50 originating from the same ERV family with syncytin-2 (derived from MER50-int) is essential for the upregulation of MFSD2A expression, promoting the conversion of hTSC to STB (Yu et al., 2023) (Supplementary Table S1). Additionally, TNFAIP2 modulating trophoblast cell fusion is also affected by the adjacent MER50-derived enhancer element (Yu et al., 2023) (Supplementary Table S1). It has been reported that LTR promoters are more active in placenta, which may be attributed to the evolutionary mechanism of endogenization as more active HERVs in reproductive system tend to be integrated into the genomes of germ cells (Cohen et al., 2009).

### 3.4 The impact of solo-LTRs on immune response

Increasing evidences demonstrate that HERVs participate in immune responses both in healthy individuals and patients with diverse diseases (Chuong et al., 2016; Lima-Junior et al., 2021; Dopkins and Nixon, 2023; Ng et al., 2023). It has been widely reported that infection of exogenous viruses (such as influenza A virus, dengue virus and retrovirus) could reactivate diverse HERVs (Srinivasachar Badarinarayan et al., 2020; Wang et al., 2020; Liu et al., 2022). For instance, LTR12C elements in the vicinity of immunity genes are activated upon HIV-1 infection of primary CD4^+^ T cells (Srinivasachar Badarinarayan et al., 2020). Accumulating evidence strongly demonstrates that two solitary LTR12C elements induced by HIV-1 modulate the expression of GBP2 and GBP5 involved in antiviral immunity (Srinivasachar Badarinarayan et al., 2020) (Supplementary Table S1). Recent studies have also revealed that STAT1 and IRF1 interact with a solitary LTR12F located upstream of HERV-K102 following IFN-γ signaling, resulting in upregulation of HERV-K102 (Russ et al., 2023). And HERV-K102 promotes the expression of genes containing interferon-stimulated response elements, which may enhance pro-inflammatory signaling in macrophages and probably other immune cells (Russ et al., 2023). Emerging evidence has showed that the integration of the primate-specific MER41 (a type of LTR in ERV1 superfamily) with STAT1-and IRF1-binding sites located in or near several genes (such as IFN-stimulated gene AIM2) contributes to primate-specific interferon responses (Chuong et al., 2016). Our further analysis found that the majority of the MER41 elements are solitary LTRs.

Therefore, it appears that solo-LTRs are beneficial gene-regulatory machinery upon viral infection. In contrast, pathological consequences such as activating oncogenic pathways in numerous cancers are widely known (Kahyo et al., 2013; Nguyen et al., 2019). So HERVs might be “double-edged swords” in human health. It has been speculated that it might be a matter of time until HERVs with detrimental effect are eventually eliminated (Chuong et al., 2017). In addition, there is another possibility that some HERVs with an adaptive role have been misregulated occasionally, thus imposing a negative side effect on the host (Chuong et al., 2017).

### 3.5 The impact of solo-LTRs on human evolution

Extremely large amounts of HERVs have affected human evolution through reserving a large repertoire of coding and non-coding sequences in the genome and became co-opted for critical physiological functions (Chen et al., 2022; Dopkins and Nixon, 2023). Solo-LTRs accounting for a high percentage of existing HERVs serve as a significant driving force for human evolution at a long-term scale as they could act as regulatory elements for genes with vital function. One of the most striking examples is the presence of a LTR12C element located upstream of ribonucleic acid export 1 (RAE1) (Supplementary Table S1), which is involved in the export of mature mRNAs from the nucleus to the cytoplasm (Jung et al., 2017). The finding also showed that the tandem repeat region (TRR) and NF-Y binding sites are critical for the regulatory activity of the LTR12C (Jung et al., 2017). Evolution analysis showed that the LTR12C element was only found in human, chimpanzee and gorilla, indicating that the LTR12C element was integrated into the primate genomes after the gorilla and orangutan lineages had diverged (Jung et al., 2017). Thus the LTRs could confer primate-specific functions through being retained in the genome.

A previous study comprehensively investigated regulatory elements derived from HERVs and uncovered a total of 794,972 TFBSs on HERV/LTR sequences (HERV-TFBSs) (Ito et al., 2017). And these TFBSs could bind proteins such as pluripotent TFs, embryonic endoderm/mesendoderm TFs, hematopoietic TFs, and the genome architectural protein CTCF (Ito et al., 2017). So HERVs including remanent LTRs may affect the human genome but also drive phenotypic evolution through altering or inducing primate-specific gene expression. Solo-LTRs are indeed a rich source of non-coding sequences, which fuel adaptive regulatory innovation during human evolution. And these LTR elements have been co-opted and lost internal coding regions, reflecting a crucial strategy in virus-host evolutionary “arms race” as LTRs could be repurposed by the host (Aswad and Katzourakis, 2012). Regulatory activities of solo-LTRs have also been observed for lncRNAs, such as lncRNA SChLAP1 and lncMER52A (Prensner et al., 2013; Wu et al., 2020). But in some situations the solo-LTR elements also participate in the formation of the first exon of the lncRNAs (Prensner et al., 2013; Wu et al., 2020). This may be attributed to transcriptional readthrough activity of LTR elements, which potentially increases the risk of activating downstream sequences.

## 4 Solo-LTR-initiated chimeric gene transcript

In previous studies, the identification of expressed HERVs was largely restricted due to the nature of repeat sequences. The application of high-throughput sequencing approaches has allowed to solve these problems, and individual HERV loci expressed in different cell types and tissues have gradually been found and elucidated. Numerous studies have demonstrated solo-LTR-initiated chimeric gene transcripts (mainly mRNAs and lncRNAs), which contribute greatly to human health and diseases (Cohen et al., 2009; Prensner et al., 2013; Gibb et al., 2015; Wiesner et al., 2015). And the solo-LTR-derived genes have been suggested as potential diagnostic and prognostic biomarkers across diverse cancer types. Therefore, studying solo-LTR-derived genes holds significant importance for tumor biology.

### 4.1 Solo-LTR-derived mRNAs

LTR elements can function as promoters and enhancers (Vargiu et al., 2016), and the 5′ and 3′ terminal R regions could provide the TSS and polyA site respectively (Gifford and Tristem, 2003). This structure of LTR elements raises the possibility that the transcriptional readthrough activity of LTRs could initiate novel transcripts. The hypothesis of forming novel transcripts including solo-LTR-derived-mRNAs has been demonstrated by increasing studies, and solo-LTR-derived-mRNAs are implicated in human health and diseases such as cancers and genetic disorders (de Wit et al., 2005; Dunn et al., 2006; Cohen et al., 2009; Wiesner et al., 2015; Babaian et al., 2016).

#### 4.1.1 Solo-LTR-derived mRNAs in cancers

**FIGURE 3:**
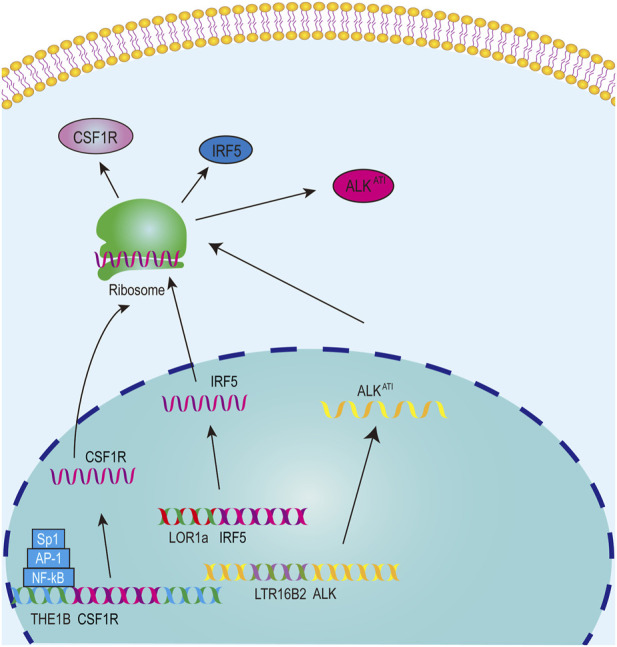
The model for solo-LTRs acting as regulatory elements.

The significance of solo-LTR-derived mRNAs has been established in various cancers, such as interferon regulatory factor-5 (IRF5) and anaplastic lymphoma kinase (ALK) isoform ALK^*ATI*^ (Wiesner et al., 2015; Babaian et al., 2016). Hodgkin’s lymphoma (HL) typically presents as a malignant disease of the lymphatic system (Townsend and Linch, 2012). The transcription factor IRF5 could regulate immune response (Ban et al., 2018). The LOR1a element, a solo-LTR from ERV1 family, is located upstream from the canonical TSS of IRF5 (Supplementary Table S1), and the hypomethylated LOR1a initiates a LOR1a-IRF5 chimeric transcript, part sequence of the first exon of which originates from LOR1a in multiple HL cell lines but not in normal B-cell controls, thus resulting in the upregulation of IRF5 in HL cell lines (Babaian et al., 2016) (Figure 3). Furthermore, an interferon regulatory factor binding element (IRFE) including target site duplication (TSD) and first few bases of LOR1a was identified, and it could control the promoter activity of the LOR1a element (Babaian et al., 2016).

ALK, a promising drug target for multiple cancers, is closely associated with the occurrence and development of cancers (Ma et al., 2023). A novel ALK isoform, namely ALK^*ATI*^, was found in approximately 11% of melanoma cases and sporadically in other cancer types but not in normal tissues (Wiesner et al., 2015). ALK^*ATI*^ can activate multiple proto-oncogenic signaling pathways and promote carcinogenesis in mouse models (Wiesner et al., 2015). The transcription of ALK^*ATI*^ starts from a LTR16B2 element of ERV3 superfamily in the 19th intron of ALK (Wiesner et al., 2015) (Figure 3, Supplementary Table S1). Luciferase report experiment showed that the LTR16B2 element could function as a promoter, and LTR16B2 also includes the TSS and part sequence of the first exon (Wiesner et al., 2015).

Colony-stimulating factor 1 receptor (CSF1R) plays a proto-oncogenic role in a diverse range of malignancies (Zhu et al., 2022) and is significantly associated with overall survival of HL patients (Steidl et al., 2012). In HL cells, the transcription of CSF1R does not initiate from the canonical CSF1R promoter but from a THE1B LTR element of the ERVL-MaLR family, located ∼ 6.2 kb upstream of the canonical promoter (Lamprecht et al., 2010) (Figure 3, Supplementary Table S1). The THE1B element contains TFBSs for NF-κB, AP-1 and Sp1, and these sites are crucial for the THE1B element to exert its promoter regulatory capacity in HL cells (Lamprecht et al., 2010) (Figure 3). The TFBSs for NF-κB, AP-1 and Sp1 are conserved in THE1 subfamily LTRs, and the expression of transcripts driven by THE1A, THE1B and THE1C in THE1 subfamily is also confirmed, indicating that THE1 activation is a widespread phenomenon in HRS cells (Lamprecht et al., 2010). The THE1B element also include the 5′ UTR sequence, which is located in the first exon. Thus these results indicate that the THE1B element initiates the transcription of CSF1R and part of the THE1B element is also transcribed into the mRNA sequence of CSF1R.

#### 4.1.2 Solo-LTR-derived mRNAs in genetic disorders

Down syndrome, characterized by an additional copy of chromosome 21, is the most common chromosomal disorder among live-born infants (Shin et al., 2009). Down syndrome critical region 4 (DSCR4) and DSCR8 (a member of the cancer-testis antigen family) share a common LTR9 promoter, which belongs to the ERV1 family (de Wit et al., 2005; Dunn et al., 2006) (Supplementary Table S1). But the transcription directions of DSCR4 and DSCR8 differ as LTR9 could act as a bidirectional promoter (Dunn et al., 2006). LTR9 exhibits superior promoter activity for DSCR4 compared to that for DSCR8 (Dunn et al., 2006). A 41 bp conserved sequence including two putative Sp1 binding sites and one putative cAMP response element (CRE) within the LTR9 element plays a crucial role in the high expression of DSCR4 and DSCR8 (Dunn et al., 2006). More importantly, part of the first exon of DSCR4 and the first exon of DSCR8 are derived from the LTR9 element. Further evolution analysis suggested that the LTR9 element also exists in the genomes of several non-human primates (Dunn et al., 2006). Therefore, the LTR9 element contributes to the transcription and formation of DSCR4 and DSCR8.

Choroideremia (CHM) is an X-linked inherited disorder resulting from mutations in gene CHM (Kellner et al., 2021). The fourth intron of gene CHM contains a solitary LTR12C element, which provides an alternative splicing site and leads to premature termination of CHM transcription (Jung et al., 2011). Thus a new CHM isoform b is produced and the last exon of isoform b is derived from the LTR12C element (Supplementary Table S1), which encodes six amino acid residues (RSTLLL) and has a new stop codon (Jung et al., 2011). High expression of CHM isoform b was identified in colon cancer and lung cancer cells as well as patient tissues of colon cancer, and CHM isoform b has been suggested as a molecular marker for cancer detection (Jung et al., 2011).

#### 4.1.3 Solo-LTR-derived mRNAs in placental development

ENTPD1 is the dominant ecto-nucleotidase of placental trophoblastic tissues (Kittel et al., 2004), and downregulated ENTPD1 could suppress trophoblast cell proliferation and invasion (Zhu et al., 2018). MER39B acts as an alternative promoter and also contributes part sequence to the first exon of ENTPD1 (Supplementary Table S1), resulting in a new transcript, which encodes a protein with a different N-terminal sequence (Makita et al., 1998; Matsumoto et al., 1999; Zhu et al., 2018). PAPPA2, the abundantly expressed protease in the human placenta, may be a useful biomarker of placental dysfunction (Wang et al., 2009). The expression of PAPPA2 is mainly triggered by a solo-LTR (MER41E) from ERV1 superfamily (Supplementary Table S1), and part sequence of the first exon of PAPPA2 is also derived from the MER41E element (Cohen et al., 2009). Similar observations have also been made for protein PTPRF, CYP19A1, IL2RB and FABP7 (Cohen et al., 2009; Lock et al., 2014) (Supplementary Table S1).

### 4.2 Solo-LTR-derived lncRNAs

LncRNAs represent the predominant class of noncoding RNAs, surpassing the number of protein-coding genes by twofold (Iyer et al., 2015; Ali et al., 2021). Many lncRNAs have been reported to act as oncogenes or tumour suppressors through abnormal expression or mutations, and thus are linked with cancer progression or suppression (Mattick et al., 2023). Although solo-LTRs are fragmented remnants generally regarded as silenced or inactivated elements, increasing studies have discovered that solo-LTRs could be transcribed as lncRNAs, which play an important role in a diverse range of biological processes (Prensner et al., 2013; Jin et al., 2019; Wu et al., 2020). Mechanistically, multiple evidences have showed that lncRNAs could engage in multilateral interactions, including targeting DNA, binding other RNAs and recruiting proteins (Mattick et al., 2023). For solo-LTR-derived lncRNAs, most results suggest that the lncRNAs are implicated in occurrence and development of various cancers through forming lncRNA-protein complex (Jin et al., 2019; Wu et al., 2020).

#### 4.2.1 LTR12C

**FIGURE 4:**
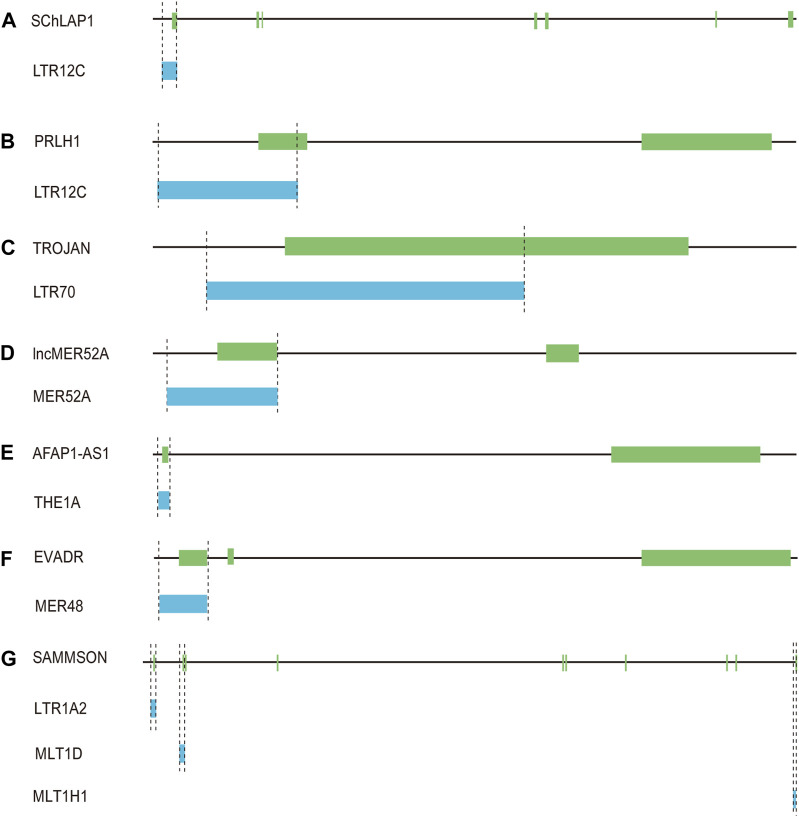
Solo-LTR-derived lncRNAs. **(A)** LTR12C-derived SChLAP1. **(B)** LTR12C-derived PRLH1. **(C)** LTR70-derived TROJAN. **(D)** MER52A-derived lncMER52A. **(E)** THE1A-derived AFAP1-AS1. **(F)** MER48-derived EVADR. **(G)** LTR1A2, MLT1D and MLT1H1-derived SAMMSON.

LTR12C elements belong to the ERV9 family, which had exhibited activity until approximately 6 million years ago (López-Sánchez et al., 2005). LTR12C sequences with greater length and higher CpG content are frequently associated with human health and disease (Babaian and Mager, 2016). As active HERV elements, solitary LTR12C sequences not only exert transcriptional regulation, but also could be processed to generate lncRNAs (Prensner et al., 2013; Babaian and Mager, 2016). In prostate cancers, lncRNA SChLAP1 is highly expressed in ∼25% of cancer tissues, and could serve as a potential target for predicting patient outcome (Prensner et al., 2013). More importantly, the transcription of SChLAP1 is driven under the upstream LTR12C region (Prensner et al., 2013) (Figure 4A; Table 1). Our further examination revealed that the first exon of SChLAP1 consists of 338 bases, of which 322 bases are located in the LTR12C element. Thus, the LTR12C sequence could influence the expression of SChLAP1, and also contribute to the first exon of SChLAP1.

**TABLE 1:** The information of solo-LTR-derived lncRNAs.

Solo-LTRs	Strand	Location	solo-LTR-derived lncRNAs	Strand of lncRNAs	Expression of lncRNAs
LTR12C	+	chr2:180691205-180692425	SChLAP1	+	Prostatic cancer
LTR12C	+	chr8:83402758-83404051	PRLH1	+	Hepatocellular carcinoma
LTR70	-	chr19:20220441-20221668	TROJAN	-	Breast cancer
MER52A	-	chr4:65241174-65242746	lncMER52A	-	Hepatocellular carcinoma
THE1A	+	chr4:7753884-7754236	AFAP1-AS1	+	Multiple human cancers
MER48	+	chr6:70394626-70395013	EVADR	+	Adenocarcinomas
LTR1A2, MLT1D and MLT1H1	+	chr3:69999501-70000000	SAMMSON	+	Multiple human cancers
	+	chr3:70012208-70012665			
	+	chr3:70389529-70389681			

**FIGURE 5:**
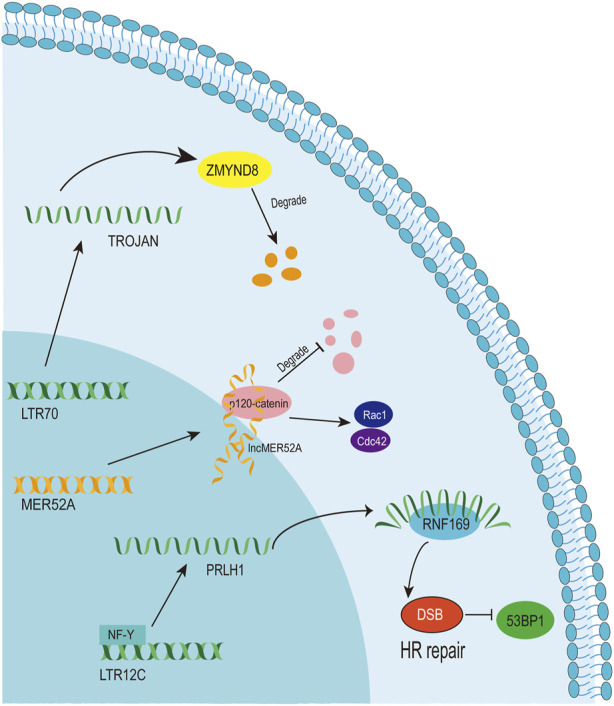
The function and mechanism of solo-LTR-derived lncRNAs.

A LTR12C-derived lncRNA called p53-regulated lncRNA for homologous recombination (HR) repair 1 (PRLH1) has also been identified (Deng et al., 2019) (Table 1). High expression of PRLH1 was found in p53-mutated hepatocellular carcinoma (HCC) samples and PRLH1 could promote the proliferation of p53-mutated HCC cells through binding to E3 ubiquitin ligase RNF169, thus supplanting 53BP1 at double-strand break sites and promoting the initiation of HR repair (Deng et al., 2019) (Figure 5). The LTR12C element is located across the promoter and the first exon of PRLH1 (Deng et al., 2019) (Figure 4B). NF-Y could promote the transcription of PRLH1 through binding two GCUUCA boxes in the LTR12C element (Deng et al., 2019). The findings of SChLAP1 and PRLH1 highlight the importance of relatively active LTR12C elements. Considering as many as 2,742 copies of LTR12C in human genome, there may be other functions of LTR12C to be revealed (Deng et al., 2019).

It has been shown that a substantial upregulation of LTR12C elements has been observed following treatment with DNA methyltransferase inhibitors (DNMTi) and histone deacetylase inhibitors (HDACi) (Brocks et al., 2017). The U3 promoter and enhancer region of LTR12 contains multiple TFBSs, including NF-Y, Sp1 and GATA2 (Yu et al., 2005). Experimental analysis has revealed that GATA2 potentially functions as a pivotal transcription factor in regulation of LTR12C expression (Brocks et al., 2017). Although it remains unclear whether the transcripts initiating from LTR12C form lncRNAs, the defective and variable sequences of LTR12C may be an indicator of lncRNA products but not mRNAs.

#### 4.2.2 LTR70

LTR70 elements are members of ERV1 superfamily. In TNBC, the transcription of LTR70 generates a primate-specific lncRNA dubbed TROJAN (Jin et al., 2019) (Figure 4C; Table 1). TROJAN exhibits high expression level in TNBC and contributes to TNBC proliferation and invasion through increasing the degradation of a metastasis-repressing factor ZMYND8 (Figure 5), thereby being linked with unfavorable prognosis of TNBC patients (Jin et al., 2019). Antisense oligonucleotides targeting TROJAN can significantly inhibit the growth and metastasis of tumors, and TROJAN has also been suggested as a potential clinical therapeutic target for TNBC (Jin et al., 2019). Furthermore, TROJAN is highly expressed in estrogen receptor-positive (ER+) breast cancer, which constitutes two-thirds of all breast cancers (Jin et al., 2020). And TROJAN is also implicated in a poor prognosis of ER + breast cancer patients (Jin et al., 2020).

Distinct from the mechanism described above, TROJAN inhibits the interaction between the NF-κB pathway repressor NKRF and RELA, a transcriptional activator of the NF-κB pathway, thereby upregulating the expression of CDK2, which ultimately reverses the resistance of ER + breast cancer to a CDK4/6 inhibitor showing the anticancer activity (Jin et al., 2020). Therefore, the existing studies illustrate the versatile and complex nature of TROJAN oncogenic role, highlighting the unexplored potential and function diversity of TROJAN.

#### 4.2.3 MER52A

A novel lncRNA termed lncMER52A with exclusive expression in HCC has been identified, and lncMER52A could promote HCC progression via stabilizing p120-catenin and activating p120-ctn/Rac1/Cdc42 axis both *in vitro* and *in vivo* (Wu et al., 2020) (Figure 5). Further analysis revealed that the expression of lncMER52A is driven by a solitary LTR named MER52A from ERV1 superfamily (Figure 4D; Table 1). The MER52A element is activated by histone modifications of H3K4me3 and H3K27ac and also bound by the transcription factor YY1 (Wu et al., 2020). The MER52A sequence not only contains a functional regulatory promoter for lncMER52A, and the initial exon of lncMER52A also originates from the 3’ terminal of the MER52A element, which is similar to the LTR12C-derived SChLAP1 in prostate cancers (Prensner et al., 2013).

#### 4.2.4 THE1A

LncRNA AFAP1-AS1 was initially identified and overexpressed in Barrett’s esophagus (BE) and esophageal adenocarcinoma (EAC) (Wu et al., 2013). AFAP1-AS1 is derived from the antisense strand of the protein-coding gene AFAP1 (Wu et al., 2013). Our further analysis showed that the first exon of AFAP1-AS1 originates from THE1A, a solitary LTR of ERVL-MaLR superfamily (Figure 4E; Table 1). In addition, the THE1A element may also serve as regulatory promoter sequence and participate in initiating the transcription of AFAP1-AS1 as the 5′ terminal region of THE1A consisting of 193 bases is located upstream of the TSS of AFAP1-AS1.

The function of AFAP1-AS1 has been extensively investigated in various cancers including EAC, lung cancer, HCC, and breast cancer (Wu et al., 2013; Deng et al., 2015; Zeng et al., 2016). In EAC cells, silencing of AFAP1-AS1 could reduce cell migration and invasion without altering the expression of AFAP1 (Wu et al., 2013). In NSCLC, expression of AFAP1-AS1 is significantly increased in tumor tissues, and knockdown of AFAP1-AS1 significantly inhibits the invasive and migration capability of lung cancer cells (Zeng et al., 2016). So AFAP1-AS1 is proposed as an independent prognostic indicator for lung cancer patients (Deng et al., 2015). In HCC, AFAP1-AS1 could also promote cancer progression (Zhang et al., 2016). In TNBC, upregulation of AFAP1-AS1 promotes epithelial-mesenchymal transition and tumorigenesis (Zhang et al., 2018). AFAP1-AS1 is implicated in carcinogenesis or cancer progression in diverse cancers, and multitudinous mechanisms involved in promoting cancer development have been reported (Wu et al., 2013; Deng et al., 2015; Zeng et al., 2016; Zhang et al., 2016; Zhang et al., 2018). For example, AFAP1-AS1 could upregulate RhoA/Rac2 signaling to promote HCC development while it activates Wnt/β-catenin pathway, resulting in tumorigenesis and cell invasion of TNBC (Zhang et al., 2016; Zhang et al., 2018).

#### 4.2.5 MER48

A specific activation of lncRNA EVADR was detected in 25%–53% of colon, rectal, lung, pancreas and stomach adenocarcinomas, and expression of EVADR is correlated with poor prognosis of adenocarcinomas patients (Gibb et al., 2015). Detailed sequence analysis revealed that the transcription of EVADR is driven by a solitary MER48, which has numerous TFBSs and a putative TATA box (Gibb et al., 2015) (Figure 4F; Table 1). MER48 can function as a bidirectional promoter through luciferase reporter assay, but only promotes the expression of EVADR *in vivo* (Ban et al., 2018). Furthermore, the MER48 element contributes 127 nucleotides to the first exon of EVADR. So the MER48 sequence plays a vital role in regulation and expression of EVADR.

#### 4.2.6 MLT1D, MLT1H1 and LTR1A2

LncRNA SAMMSON was initially discovered as an oncogene in skin melanoma and could disrupt vital mitochondrial functions in a cancer-cell-specific manner (Leucci et al., 2016). Numerous studies have further shown that SAMMSON is upregulated in several cancers including melanoma, breast cancer, glioblastoma and liver cancer and has an oncogenic role in multiple cancers (Ghasemian et al., 2023). Up to 28 transcripts of SAMMSON are produced through alternative splicing (Ghasemian et al., 2023), and many solo-LTRs contribute to the exonic formation of the transcripts. For instance, the canonical transcript of SAMMSON is composed of ten exons. And through inspection we found that the first two and the tenth exons include solo-LTR sequences (Figure 4G; Table 1). Among the three exons, the total second exon (242 bases) consists of the sequence from the MLT1D element, which belongs to old ERVL-MaLR superfamily (Figure 4G). And about 38% sequence of the tenth exon (286 bases) is derived from MLT1H1, also a solo-LTR element from ERVL-MaLR (Figure 4G). The first exon has only 22 bases, which also comes from a solo-LTR, referred as LTR1A2 from ERV1 superfamily (Figure 4G). Therefore, these results raise the possibility that MLT1D, MLT1H1 and LTR1A2 may be the crucial components of SAMMSON but also play an important role in the biological function of SAMMSON.

## 5 Conclusion

Solo-LTRs make up a large majority of the HERVs persisting in the human genome. Previous studies have shown that some of the many thousands of solo-LTRs are involved in various biological processes by acting as promoters/enhancers. In this review, we summarized the recent discoveries about the functions of a few solo-LTRs and the chimeric genes (especially lncRNAs) stemming from them and proximal sequences, thereby gradually uncovering the mechanisms and important functions of these few solo-LTRs. And the different pathways also reflect the complexity of solo-LTR-derived genes (Figure 5), which could affect tumor biology greatly. This review aims to highlight that solo-LTRs can alter transcription regulation of adjacent genes by providing enhancers and promoters and also shape the human transcriptome through generating lncRNAs. Although the functions of small quantity of solo-LTRs have been revealed and the precise mechanism and effect of a bulk of solo-LTRs activation remains to be uncovered, there is no doubt that solo-LTRs play a pivotal role in human health and disease. Further exploration to unveil the pathological processes and underlying mechanisms of solo-LTRs is urgently needed.
