# Supplementary material for: Endogenous retroviral solo-LTRs in human genome
Source: Front Genet. 2024 Mar 28;15:1358078. doi: 10.3389/fgene.2024.1358078 (PMC11007075; doi:10.3389/fgene.2024.1358078)
Supplement: Supplementary file 1 [file Table1.docx]

**Table S1.** The information of solo-LTRs and adjacent genes mentioned in the paper

| Solo-LTRs | Strand | Location | Adjacent genes to solo-LTRs | Strand of adjacent genes | Expression of adjacent genes |
| --- | --- | --- | --- | --- | --- |
|  |  |  |  |  |  |
| MER21A | + | chr3:187021812-187022350 | ST6GAL1 | + | B cells |
| LTR12C | - | chr11:5293260-5294954 | β-globin gene locus | - | Erythroblasts |
| LTR5HS | - | chr12:51454287-51455254 | SLC4A8 | + | Breast cancer |
| LTR5HS | + | chr2:27459979-27460946 | IFT172 | - | Breast cancer |
| LTR5_HS | - | chrX:135302739-135303701 | ZNF75D | - | Lung cancer |
| LTR8B | + | chr19:43182008-43182757 | PSG5 | - | Placenta |
| LTR10A | + | chr9:127848841-127849388 | ENG | - | Placenta |
| LTR10A | + | chr5:150109903-150110464 | CSF1R | - | Placenta |
| MER50 | - | chr1:39941718-39942244 | MFSD2A | + | Placenta |
| MER50 | +  + | chr14:103117852-103118043,  chr14:103116457-103117027 | TNFAIP2 | + | Placenta |
| LTR12C | - | chr1:89127019-89128609 | GBP2 | - | T cells |
| LTR12C | - | chr1 :89272453-89273890 | GBP5 | - | T cells |
| LTR12C | + | chr20:57348214-57349645 | RAE1 | + | Multiple human cell lines |
| LOR1a | + | chr7:128936859-128937097 | IRF5 | + | Hodgkin lymphoma |
| LTR16B2 | - | chr2:29223783-29224196 | ALK*^ATI^* | - | Multiple human cancers |
| THE1B | - | chr5:150092453-150092809 | CSF1R | - | Hodgkin lymphoma |
| LTR9 | - | chr21:38121187-38121599 | DSCR4 | - | Prostate, thymus, muscle, testis and placenta |
| LTR9 | - | chr21:38121187-38121599 | DSCR8 | + | Prostate, brain, kidney, bone marrow, muscle, testis and placenta |
| LTR12C | - | chrX:85971257-85972921 | CHM | - | Colon cancer and lung cancers |
| MER39B | + | chr10:95710950-95711500 | ENTPD1 | + | Placenta |
| MER41E | + | chr1 :176462795-176463370 | PAPPA2 | + | Placenta |
| LTR16A2 | + | chr1:43521788-43522217 | PTPRF | + | Hodgkin lymphoma-derived cellS and primary  mediastinal large B-cell lymphoma-derived cells |
| MER21A | - | chr15 :51338476-51338990 | CYP19A1 | - | Placenta |
| THE1D | - | chr22:37174876-37175252 | IL2RB | - | Placenta |
| LTR2 | - | chr6:122748805-122749262 | FABP7 | + | Diffuse large B-cell  lymphoma |
